# Determinants of Low Coverage of the Free Surgical Care Programme for Trachomatous Trichiasis in Rural Guinea in 2022

**DOI:** 10.3390/tropicalmed9100239

**Published:** 2024-10-11

**Authors:** Lamine Lamah, Delphin Kolié, Akoi Zoumanigui, Nouhou Konkouré Diallo, Mamadou Camara, Hawa Manet, Tamba Mina Millimouno, Bienvenu Salim Camara, Aissata Tounkara, Alexandre Delamou

**Affiliations:** 1 Helen Keller International, Conakry P.O. Box 6050, Guinea; 2 National Training and Research Centre in Rural Health of Maferinyah, Forécariah P.O. Box 2649, Guinea; dkolie@maferinyah.org (D.K.); hawa@maferinyah.org (H.M.); mina@maferinyah.org (T.M.M.); bscamara@maferinyah.org (B.S.C.); adelamou@cea-pcmt.org (A.D.); 3 African Center of Excellence for the Prevention and Control of Communicable Diseases, University of Conakry, Conakry P.O. Box 1017, Guinea; atounkara@cea-pcmt.org; 4 Programme National de Lutte Contre les Maladies Tropicales Négligées à Chimiothérapie Préventive, Conakry P.O. Box 585, Guinea; drzoumaniguipnlmtn@gmail.com (A.Z.); dnouhoufr@yahoo.fr (N.K.D.); 5 Programme National de Lutte Contre les Maladies Tropicales Négligées à Prise en Charge des Cas, Conakry P.O. Box 585, Guinea; mamadycamarafr@yahoo.fr

**Keywords:** trachomatous trichiasis, free surgical care, challenges, rural areas, Guinea

## Abstract

This study aimed to describe the experiences of healthcare personnel and patients in the organization of free surgical campaigns and care for trachomatous trichiasis in the health district of Siguiri in Guinea, including challenges experienced in providing surgical care. This was an explanatory qualitative study conducted in 2022 in the health district of Siguiri. A total of 20 participants were interviewed including patients (*n* = 7; 35%), community health workers (*n* = 4; 20%), health services managers, and healthcare providers (*n* = 8; 40%). Two main data collection technics were used: documentary review and in-depth individual interviews. All interviews were transcribed and manually coded using an Excel extraction spreadsheet. Data were analysed using inductive and deductive approaches. The results showed several organizational, structural, and community challenges that underlined the low surgical coverage of trachomatous trichiasis in the health district of Siguiri. Organizational challenges included the low involvement of local actors in planning activities, the limited timeframe of the campaigns, and the lack of logistics for activities supervision and patients’ transportation to surgery sites. Structural challenges included the inadequacy of health centres to provide surgical services, poor health infrastructures, and sanitation conditions in some areas. Individual challenges included remoteness of surgical sites and costs associated with services provision including medicines. Community challenges included fear of surgery and the coincidence of the campaigns with agricultural and mining activities. The results of the study call on the national neglected tropical disease program and its partners to adopt and promote micro-planning of trachomatous trichiasis surgical activities, with the effective participation of local stakeholders in endemic health districts. They should also envision integrating the management of surgical treatment including costs associated with care (transportation, food, rehabilitation support for patients who have undergone surgery) and complications of surgical procedures for an expansion of the trachomatous trichiasis free surgical care coverage in endemic health districts in Guinea.

## 1. Introduction

Trachoma is a bacterial eye infection caused by Chlamydia trachomatis. It constitutes a public health problem in 44 countries worldwide, mainly in rural areas of Africa, Asia, Central and South America, Australia, and the Middle East [1]. It is responsible for visual blindness in about 2 million people [1]. Blindness due to trachoma is irreversible and represents about 1.4% of total blindness cases worldwide [1]. In endemic regions, 60 to 90% of active trachoma (inflammatory) cases affect children aged 1–9 years, predisposing them to repeated infections and eye damage with an increased risk of compromising their future [1]. The economic impact of trachoma is considerable because of the loss of productivity caused by the irreversible blindness it causes [1]. This economic loss is estimated to be between 3 and 5 billion US dollars (USD) per year, and as much as 8 billion US dollars when trachomatous trichiasis (the complication of trachoma) is included [1,2].

The recent World Health Assembly resolution aims to eliminate trachoma by 2030 through the new roadmap approved in 2020 [1]. To achieve this, four main actions, outlined in the “SAFE” strategy, are recommended [1]. These include surgery to treat trachomatous trichiasis, antibiotic therapy to treat the infection, facial cleanliness, and environmental improvements to curb the disease transmission chain [1]. However, trachoma surgery is only indicated in the stage of trachomatous trichiasis (TT), and it prevents progression to blindness or corneal opacity [3]. For the first two stages of the disease (follicular and intense inflammatory trachoma respectively), antibiotic therapy and personal and environmental hygiene are recommended [3].

The surgical coverage rate of trachoma remains low in sub-Saharan Africa despite the existence of free care programmes. Coverage is estimated to be between 44 and 66% according to recent studies [4,5]. Few studies document the reasons for the low utilization of trachoma free surgical care programmes in sub-Saharan Africa. However, Bowman et al. in the Gambia reported better trachoma surgical coverage in programmes offering community-based services compared to those based in health facilities [4]. Other studies in Malawi, Tanzania, and Ethiopia have reported that the costs associated with care (transportation, hospital fees, etc.) and the short period of the free surgical care campaigns negatively influence the utilization of trachoma surgical care services [6,7,8]. Churko et al. in Ethiopia have also shown that fear of surgery and negative perceptions of eyesight, patients’ beliefs in supernatural powers, limited motivation of surgeons, and lack of logistics are the main factors underlying the low utilization of trachoma surgical care programmes [8].

In Guinea, trachoma is a public health problem due to its frequency and geographic distribution. A mapping exercise conducted between 2011 and 2016 identified 18 endemic health districts and indicated that nearly 33,000 people needed surgical care [9]. However, the health districts most affected by TT are in the region of Kankan (Kankan, Kouroussa, and Siguiri) and Faranah (Dabola, Dinguiraye, Faranah, and Kissidougou); namely, 69.6% (22,782/32,737) of the total cases in Guinea [9].

To accelerate the control and elimination of trachoma in Guinea, the national programme for neglected tropical diseases control initiated in 2013 the free surgical care programme in endemic health districts in the country. This free surgical care programme aimed to manage nearly 33,000 cases of TT by 2020 [9]. However, the objectives of the programme were not achieved, since in June 2021, only 12% of the expected patients had benefitted from surgical care. In the region of Kankan, and for the same period, only 696 people had benefitted from surgical care for TT. In the health district of Siguiri, 183 (9%) patients had undergone TT surgical care in 2021, out of the 2000 cases expected to be managed in the same year.

This study was therefore initiated to better understand the determinants of the low coverage of TT surgical care services in rural Guinea. Specifically, this study aimed to describe the experience of stakeholders in the Siguiri health district in organizing TT surgical campaigns and to explore the challenges faced by health services managers, healthcare providers, and communities in the provision of free surgical care for TT. Such a study could provide policymakers with relevant information to improve the coverage of surgical care for TT in Guinea and therefore contribute to the achievement of disease elimination objectives by 2030.

## 2. Materials and Methods

### 2.1. Type of Study

This was a qualitative explanatory study conducted in the health district of Siguiri in Guinea in 2022.

### 2.2. Study Setting

#### 2.2.1. General Setting

Guinea is a West African country with a population of 13 million in 2022, the majority of whom live in rural areas and below the poverty line [10].

The administrative structure of Guinea is composed of eight regions: Boké, Faranah, Kankan, Kindia, Labé, Mamou, N’Zérékoré, and the capital Conakry. In total, there are five communes in Conakry and 33 districts, or 38 health districts throughout the country. Each district is divided into urban and rural communes (or municipalities).

The health system is based on a three-tiered pyramid: the primary level, which includes health posts and centres; the secondary level, which includes regional and district hospitals and communal medical centres (exceptionally located in Conakry, the capital); and the tertiary level, which includes three national hospitals. In 2019, the country had a ratio of about one skilled health worker per 10,000 inhabitants, unevenly distributed between rural and urban areas, to the disadvantage of the former [11].

#### 2.2.2. Specific Setting

**Figure 1:**
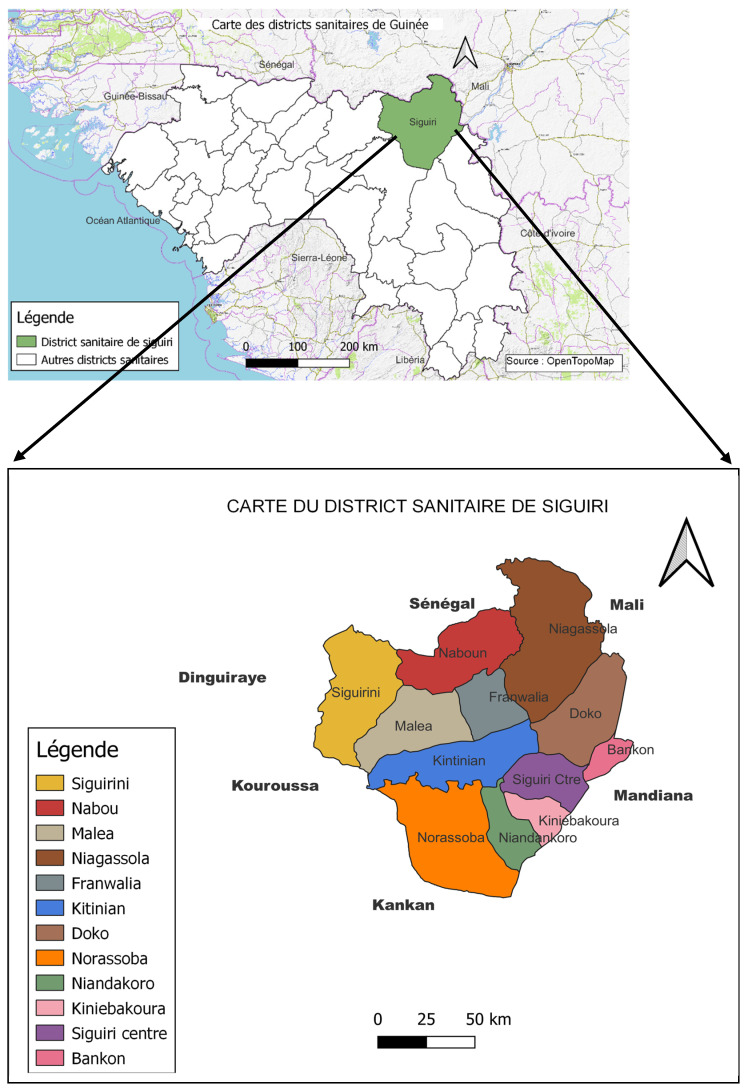
Study sites.

The study was conducted in the Siguiri rural health district located in the Kankan region. It had an estimated population of 855,494 inhabitants in 2022 [10] (see Figure 1).

The Siguiri health district has a prefectural hospital, 12 rural health centres, and four urban health centres.

The mapping carried out in 2013 showed that the Siguiri district hosted 5.1% of total TT cases in the country, with a forecast of 7700 cases to undergo surgical care [9]. After three rounds of mass treatment with Azithromycin and Tetracycline ophthalmic ointment and the organization of several TT surgical campaigns, an impact assessment survey was conducted in 2017 and a surveillance survey in 2021. These two studies concluded that the prevalence of TT in the health district of Siguiri was 1% (0.39 to 1.57) and 4.4% (2.68 to 6.35), respectively. This indicated that despite surgical efforts, the prevalence of TT had not substantially decreased in this health district, compared to the baseline prevalence. Moreover, despite free surgical care for TT, less than 10% of the planned TT cases benefitted from surgical care in the Siguiri health district. There is little information on the reasons for this low surgical coverage of TT despite the existence of the free surgical care programme in the country.

#### 2.2.3. Management of Trachoma in Guinea

In Guinea, strategies for trachoma control are aligned with the WHO recommendations, and these are carried out through the “SAFE” strategy. This involves surgery to treat TT; antibiotic therapy to treat the infection; cleaning the face; and changing the environment to stop the chain of transmission of the disease [12,13]. As of 2022, the national program stopped antibiotic treatment in the 16 health districts that are endemic for trachoma after more than one round of treatment per year. The surgical care for TT is carried out essentially through surgical campaigns [10]. The free surgical care for TT is performed by ophthalmic technicians with the financial support of partners in the endemic health districts of Kankan, Siguiri, Mandiana, Kouroussa, Dinguiraye, and Beyla.

Health system personnel in charge of implementing the free surgical care program for TT include staff of the national NTD control program (main role = campaigns planning), local health workers (main role = supervision), community workers (main role = awareness raising, screening), and ophthalmic technicians (main role = performing the surgery).

This free program began with the training of community health workers on the symptoms of trachoma and the techniques for its detection. Following this, community health workers were supported to organize community awareness-raising sessions within the communities. These sessions had three objectives including informing the community members about the clinical manifestations of trachoma, its consequences, and the importance of early screening and treatment. Community health workers perform household visits to examine eyes for signs of trachoma trichiasis, such as eyelashes rubbing against the eyeball. Cases detected in this process are therefore referred to treatment sites or camps for confirmation and management.

In 2023, the TT surgery program was integrated into the routine healthcare system, following the development of a transition plan with the Siguiri health district management teams.

In the Siguiri health district, free surgical care for TT is provided through advanced surgical campaigns and surgical camps. The advanced surgical care strategy for TT is realized through mobile teams that travel from village to village to detect and manage TT cases. Meanwhile, the surgical camps represent a fixed strategy and are carried out in the following manner: sensitization of the population to gain acceptance for surgery, transportation of all cases detected by community agents to the sites selected for surgery, triage of detected cases, examination and registration of trichiasis patients, consent for surgery, admission of patients to the operating room, and immediate and three- to six-month post-operative follow-up.

### 2.3. Study Participants and Recruitment

Four groups of participants were considered and included in this study: health district and health services managers; healthcare providers including community health workers; community leaders; and patients with TT (be beneficiaries of surgical care for TT or not). These participants were recruited on a purposive basis which considered the maximum variation in their socio-demographic characteristics, their involvement in the organization of the surgical campaigns and the TT management process in Siguiri (health district and health service managers), their role in the provision of care (healthcare providers), and their perceptions of the health services (patients). The recruitment of participants also complied with the principle of theoretical saturation, meaning that participants were interviewed until no new information emerged from the interviews.

### 2.4. Data Collection

Two main data collection techniques were used: document reviews and in-depth individual interviews (IDIs). Reports of activities or campaigns, missions, and supervision of TT surgery in the Siguiri health district were collected from district and health service managers in parallel with the conduct of the IDIs. The IDI guides were pre-tested before data collection. IDIs with patients and community health workers were conducted in the local language. The IDIs were digitally recorded. A total of 20 IDIs were conducted.

### 2.5. Data Analysis

The IDIs were literally transcribed into French. The focus of the analysis was on the observation notes, the transcripts of the IDIs, and the content of the TT surgical program documents. All the transcribed interviews and program documents were manually coded using an Excel extraction grid. A thematic content analysis was conducted using an inductive approach based on the data collected (empirical data).

### 2.6. Ethical Consideration

The research protocol for this study was approved by the National Ethics Committee for Health Research of Guinea (No: L-110-CNERS-22).

## 3. Results

### 3.1. Profile of Respondents

A total of 20 participants were interviewed, including patients (*n* = 7; 35%), community workers (*n* = 4; 20%), service managers, and providers (*n* = 8; 40%) (Table 1).

### 3.2. Description of the Organization of the Free Surgical Care of Trachomatous Trichiasis

The programme documents and the IDIs showed that the organization of the surgical care for trachomatous trichiasis (TT) followed three main stages or phases: the surgical campaign phase (planning of activities), the per-operative phase (the performance of the surgical procedures), and the post-operative phase (the follow-up of the operated patients).

#### 3.2.1. Activity Planning Phase

According to respondents, the planning activities for TT management were the organization of preparatory meetings, the training of community health workers for the detection of TT cases, and the training (or upgrading) of TT surgeons.

Regarding the organization of preparatory meetings, several respondents mentioned that these meetings followed a top-down logic, starting from the central level, which defined the guidelines for the campaigns, to the health centre level, which was responsible for implementation. For example, at the central level, the preparatory meetings would aim to adopt a timeline of activities, designate national supervision teams, estimate the costs of activities, and advocate with partners to mobilize the resources necessary for the effective implementation of activities. In addition, at the health centre level, the IDIs revealed that preparatory meetings were often organized on the eve of the surgical campaigns and brought together the heads of sectors, the chairpersons of the health and hygiene committees, and the health centre staff. These preparatory meetings were essentially aimed at informing and mobilizing community personnel (community leaders, community agents, etc.) to ensure the success of the surgical campaigns.

“…there was a series of meetings that brought together the national, regional, district level, but also the actors at the level of the health centers.”

**IDI 06, head of service, medical doctor, Male.**

As for the training (or retraining) of surgeons, it lasted three days and included two phases: the theoretical phase and the practical phase. These phases were preceded by the selection, at the national level, of senior technicians in ophthalmology to act as surgeons. In the Siguiri health district, this training was conducted at the prefectural hospital with the technical support of national trainers assisted by an international expert. However, respondents pointed out that the latest training sessions were entirely facilitated by national trainers.

“…[We also] received an international expert who came with the national level to train surgeons [senior ophthalmic technicians] from other prefectures and regions … The surgeons [senior ophthalmic technicians] are trained for three days.”

**IDI 06, head of service, medical doctor, Male.**

Finally, according to the respondents, the training of community relays was carried out jointly by the trained surgeons and the heads of health centres with the technical, financial, and material support of the district management teams and the national program for the control of neglected tropical diseases with preventive chemotherapy. The program’s support consisted of providing the district management teams with image boards on the TT, as well as equipment (torches, case counting forms, etc.) for case detection. Also, during this training, emphasis was placed on the clinical manifestations of the disease as well as the methods of eye examination.

“…We’ve been equipped with instruments that allow us to do the job … the flashlight and the pictures that allow us to detect whether the person has this disease or not….”

**EIA 19, community worker, male.**

“…The hair from the eye [falls] into the eyes; that’s what causes tearing… If it’s not taken care of quickly, it can lead to vision problems.”

**IDI 02, deputy health centre, Nurse, Male.**

#### 3.2.2. Carrying Out the Activities

From the point of view of several respondents, the implementation of TT surgical management activities was preceded by the national program for NTD control communication of the activity schedule and the provision of financial resources to the health district team.

At the district team level, the implementation of activities consisted essentially of defining the surgical sites (or camps) and setting up the campaign supervision teams. According to the respondents, the definition of the surgical sites depended on the number of reported TT cases during the case detection (planning period). According to the respondents, the health district management team participated in social mobilization through the dissemination of radio announcements and provided logistical support to the health centres for the transportation of patients.

At the health centre level, the implementation of activities included, according to the respondents, the reorganization of operating rooms, the sensitization of TT cases (using previously completed enumeration forms), the referral or transportation of cases to surgery sites (or camps), the sorting of patients by surgeons, the counselling of cases, and the actual surgery. According to several respondents, the actual surgery was performed by a mobile team that travelled from one surgical site (or camp) to another. The duration of the surgery campaign for each site varied from 3 to 5 days.

“…There are no operating rooms in the health centers to do surgery well… We were forced to turn a consultation room [in our health center] into an operating room to do [perform] the procedures.”

**IDI 05, health centre manager, nurse, Male.**

#### 3.2.3. Post-Operative Follow-Up

According to the respondents, patients who underwent TT were discharged the same day after the operation.

From the point of view of several respondents, post-operative follow-up of patients was scheduled on the first day (the day after the operation, for the dressing) and on the seventh day (for the removal of sutures). On the seventh day, according to the participants, a counselling session was also conducted on the attitude that the patient should adopt, in particular, the cessation of all activities for three months to avoid complications. Six months after the operation, a follow-up visit of the operated patients was expected to examine the progress of the patients and to diagnose possible complications. Cases of complications recorded in the health centres were referred to the ophthalmology unit of the Siguiri prefectural hospital for treatment.

### 3.3. Challenges Related to the Free Management of Trachomatous Trichiasis

Several challenges (or barriers) emerged from the IATs that would have hindered the effective implementation of the free TT program interventions. Depending on the level of the health system where these challenges occurred, we grouped them into four main categories: organizational challenges, health facility challenges (or structural challenges), community challenges, and individual challenges.

#### 3.3.1. Organizational Challenges

Four main challenges emerged from the EIAs concerning the organization of TT surgical management activities in the Siguiri health district. These were (1) the weak involvement of local personnel (health district managers, health facility managers, community leaders) in the planning of the surgical campaign, (2) the late communication of the chronogram of the surgical campaigns, (3) the time limit for the surgical campaign itself, and (4) the insufficient logistical support of the district team for the supervision of the activities of the surgical campaigns, a challenge that influenced the organization of TT management activities. Regarding the first challenge, respondents reported that the planning of the surgical campaign, including the estimation of financial, material, and human resource needs and the establishment of the duration and timing of the surgery, was carried out by central level personnel (national program and partners). According to the respondents, this weak involvement of local personnel in the planning of the campaigns led to interference with activities at the district level, to shortcomings in the preparation of the campaigns (omission of the contents of surgical boxes, sterilization materials, etc.), and sometimes even to the frustration of certain health service managers whose staff was demobilized for several weeks without their consent. According to other participants, this unilateral planning of surgical campaign activities did not allow for the consideration of local realities, such as the agricultural and mining activities of the communities, for effective patient mobilization.

“…When the management team came, the first day there was a delay; the surgical equipment was not sterilized even though there was no equipment for steam sterilization….”

**IDI 05, health centre manager, Nurse, Male.**

In relation to the second challenge, respondents mentioned a delay in communicating the timing of the surgical campaigns, as one respondent testified:

“…There have been difficulties because the information came late to the community level. For such a campaign, we must pass the information two (2) weeks in advance because people have to go to the health centres here.”

**IDI 01, head of services, medical doctor, Male.**

About the third challenge, a health worker interviewed in Doko (surgical site) reported that “the TT surgery campaigns last only 3–5 days”, which, according to the same respondent, “is insufficient to take care of all the patients in a health area”. However, an official from another health centre reported that “at the end of the time required for surgeons to be in a health facility, patients are often redirected to another site for treatment”. Although some respondents appreciated this coordination between surgical sites (or camps) for the management of patients, others felt that this “generates frustration for patients who can sometimes wait 3 days before being operated on” and this would lead to the retraction of many patients from pursuing care for TT.

Finally, about the fourth challenge, the respondents maintained that the lack of logistical means would limit the capacity of district personnel to support certain management teams, particularly in the transportation of patients between surgical sites, and in the identification and resolution in the field of failures inherent in the organization of these campaigns.

#### 3.3.2. Structural Challenges

The analysis of the EIAs revealed five main challenges to the management of TT related to health facilities. These challenges included (1) the inadequacy of health centres to provide surgical services, (2) the dilapidated state of some health facilities, (3) the lack of good quality light, (4) the shortage of health personnel, and (5) the remoteness of health centres from certain communities.

Regarding the first challenge, although several respondents claimed that some health centre facilities (notably consultation rooms) had been refurbished for the practice of TT surgery, the EIAs showed that these rooms were not adequate for the practice of surgery due to, among other things, the lack of operating tables and comfortable chairs for surgery. These precarious working conditions, according to the respondents, influenced the surgeons’ working hours. Other respondents also claimed that some surgical facilities were set up on the day the campaign was launched, which they felt reduced the number of patients they had to care for on a daily basis.

About the second and third challenges, which concerned the dilapidated and unhygienic state of certain health facilities, respondents said that surgical teams were forced to relocate from certain management sites for fear of exposing patients to nosocomial infections.

Respondents also noted that some health centres (or care sites) were far from the populations targeted by these campaigns, including patients previously screened and identified as having TT. This situation, according to some respondents, made it impossible to effectively manage some cases of trachomatous trichiasis in the absence of travel arrangements by the district teams.

“…agents were recruited in the villages to detect cases [during the campaigns]. They did a week’s work; they take their names and phone numbers in a register. There was the surgical camp [site] in Kintinia, but because of the distance the patients had to travel to another surgical camp [site] in Didi… The team [of surgeons] in Didi had to exceptionally grant one additional day, making it 2-day program for [the care of] the patients [living around] Didi.”

**IDI 18, deputy head of health centre, Nurse, Male.**

Finally, about the fourth challenge, respondents highlighted the lack of qualified health personnel at the local level as a significant challenge to the implementation of TT management campaigns. Some respondents linked this shortage of trained personnel to screening errors by health workers during preparatory activities for surgery. The following is a verbatim report from a health centre manager who was interviewed on this subject.

“…They need to strengthen the surgical team and the people who come must also be qualified. If we recruit trainees and send them to the field, they will try to operate but there will be many recurrences.”

**IDI 02, deputy head of health centre, Nurse, Male.**

#### 3.3.3. Community Challenges

The EIAs revealed that two (2) main challenges influenced the use of surgical services by patients with TT. These challenges included the negative perception of the community towards surgery and the conflict of surgery campaigns with agricultural and/or mining activities.

Regarding the first challenge, several respondents noted that communities perceived eye surgery as a last resort to care that they would only adopt after exhausting all other usual avenues of care. These respondents linked this community attitude to either a fear of surgery or its consequences (recurrence).

This community perception influenced the care-seeking behaviour of many community members, who were afraid of the judgements of their families and friends in seeking TT surgical services. This negative influence of the community on the patients would be supported by the lack of a mechanism for managing complications (recurrences) as testified to by this person from the district management team.

“…I refused because my eyes were sensitive, and I don’t want to go there because I don’t want to damage my eyes, because they couldn’t do anything the first time, and they won’t be able to do anything this time.”

**IDI 07, beneficiary patient, female.**

“…we received a patient who had a postoperative bud, it is us who manage these complications although we are not associated with the realization of activities … It is the responsibility of the service since we can no longer ask them for money because they were operated on free of charge, wanting to tell them here to give the money so that we can manage your complication, this causes a problem in the understanding of the patient, he will not be able to understand; why at the primary level you operated on us free of charge and now there is a complication and you are asking us for money.”

**IDI 03, head of service, medical doctor, Male.**

Finally, about the second challenge, several respondents reported that surgical management campaigns occurred at the same time as agricultural and mining activities. Some respondents argued that the lack of integrated management (lack of food, no transportation back to the community and failure of health personnel to accompany the operated patients) hindered respondents’ willingness to use surgical services during TT management campaigns. In addition, other respondents also pointed out that the use of TT services required several weeks of rest and compliance after surgery. All of these factors negatively influenced patient participation in TT surgery management campaigns, as shown below.

“…When they came [for the surgery campaign], we were very busy in the mine… We could not get back to Doko [surgery site] in time. When we were ready to go, they informed us that the doctors had left.”

**IDI 11, non-beneficiary patient, female.**

#### 3.3.4. Individual Challenges

The EIAs revealed that three (3) main challenges influenced the use of surgical services for patients with TT. These challenges included (1) distance to care sites, (2) costs associated with TT surgical care, and (3) underlying medical conditions such as diabetes. The following verbatims summarize the individual factors influencing TT patients’ use of surgical services.

“…we had recruited agents in the villages who did case detection [during the campaigns]. They did a week’s work; they take their names and phone numbers in a register. There was the surgical camp [site] in Kintinia, but because of the distance the patients had to travel, another surgical camp [site] was opened in Didi... The team [of surgeons] undertook a two-day program for [the care of] the patients [living in the vicinity] of Didi.”

**IDI 18, deputy head of health centre, nurse, Male.**

“…since the operation things have not changed with my eyes, I feel the same symptoms as before. My operation was not successful.”

**IDI 11, beneficiary patient, female.**

#### 3.3.5. Summary of Challenges

**Figure 2:**
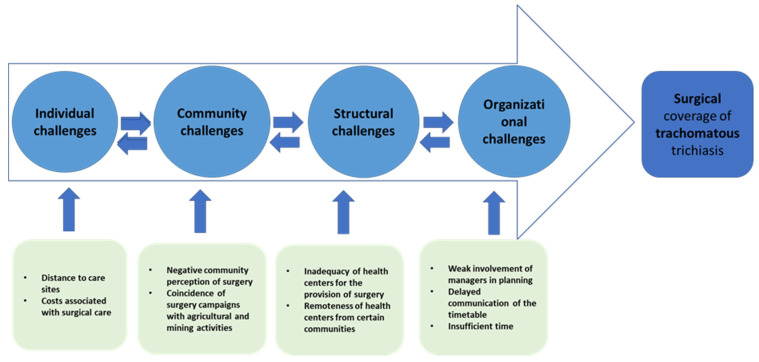
Summary of individual, community, structural, and organizational challenges influencing surgical coverage of trachomatous trichiasis in Siguiri Health District, Guinea, 2022.

The various challenges at the individual, community, structural, and organizational levels are summarized and shown in Figure 2.

## 4. Discussion

This study highlighted several organizational, structural, community, and individual challenges that determined the low surgical coverage of TT in the Siguiri health district. Organizational challenges included the weak involvement of local managers in the planning of surgical campaigns, leading to interference of activities with implications for the demobilization of routine health services staff in favour of the campaigns, sensitization and mobilization of patients and communities, and delays in the effective start of surgical campaigns. Our results also showed that the inadequacy of health centres for the provision of surgical services, the lack of qualified health personnel, and the remoteness of health facilities from certain communities influenced the management of patients during the TT surgical campaigns. Finally, the clashing of surgery campaigns with the agricultural and mining activities of the communities, and the lack of integrated management of trichiasis surgery, including food, transportation, and costs associated with surgical complications management, constituted challenges for community adherence to trachomatous trichiasis management campaigns in the health district of Siguiri in Guinea.

This study combined a literature review with individual interviews, thereby strengthening the internal validity of the data collected through triangulation. In addition, because of the researchers’ familiarity with the context and the diversification of the study participants, transferability of the results of this research to other health districts in Guinea is plausible. However, it should be noted that very few patients were included in this study, due to difficulties in locating them. This may impact data saturation on individual challenges in accessing trachomatous trichiasis services.

Our study showed that the medical costs associated with care (transportation, food, etc.) were important barriers to patients’ access to free trachomatous trichiasis surgery in the health district of Siguiri, Guinea. Rajak et al., in their study of 2591 surgical patients in Ethiopia, found that 43% and 36% of patients, respectively, were unable to reach previous surgical services due to financial difficulties and lack of an escort to the management sites [14]. In Egypt, Moussa et al., in their study evaluating the impact of TT surgery campaigns, reported that the absence of warning signs and the costs associated with care prevented 17% and 13% of patients, respectively, from accessing free TT surgery services [15]. Other researchers in Ethiopia reported that the distance to the care sites and the burden of household chores influenced access to free surgical care [15]. Our results reflected both low financial and decision-making autonomy of trachomatous trichiasis patients. This was even more evident as trachomatous trichiasis mainly affects vulnerable people such as the poor and women [16,17].

Our study suggests the need to adopt a holistic approach to the management of TT patients in Guinea, including the payment of transportation costs (round trip), food, and livelihood for the recovery period following the operation. Such an initiative could promote community adherence to the TT surgery campaigns and compliance with the surgical care.

We noted that fear of surgery negatively influenced community adherence to free TT services in the health district of Siguiri. Gupta et al. in Tanzania also reported that fear of surgery, misconceptions about recovery time, unavailability of family members to care for patients during the recovery period, and the campaign period all contributed to the patient’s refusal to undergo surgery [7]. This finding highlights the persistence of misconceptions about TT surgery despite multiple awareness campaigns in affected communities in Guinea. This finding is particularly evident as this study found that patients who underwent trichiasis surgery developed post-operative complications, including pain and swelling.

These complications reported in our study would have occurred outside of the campaign periods, thus forcing these patients to resort to specialized ophthalmology services available only at the prefectural hospital. However, based on our field experience, the surgeons involved in the trichiasis campaign come, in most cases, from outside the Siguiri health district. In addition, the direct costs of managing the complications of trichiasis surgery at the prefectural hospital are not covered by the free program. This implies that the management of complications generates additional direct and indirect costs for patients, and this could lead to a feeling of abandonment on the part of health care providers, as well as being a source of fear and refusal to accept future trichiasis surgery campaigns by the surrounding communities. This finding reiterates the need for program implementers to integrate the direct (drugs, consultation fees, etc.) and indirect (transportation, food, etc.) costs of complications from TT surgery. Another proposal would be to form a pool of surgeons for each endemic district. It would also be important to consider training, motivating, and retaining health centre nurses for post-operative follow-up of patients in endemic health districts in Guinea. Such interventions could help sustain the gains of the free care program, particularly through the gradual integration of trichiasis management into the routine services of health facilities. Our study showed that the low involvement of local authorities in the planning of TT surgery campaigns was a major challenge to the successful organization of surgery campaigns. This finding is not only applicable to the trachoma surgery program but also to a wide range of vertical program interventions, particularly in resource-limited countries where the acquisition of funding for an intervention is often perceived by the personnel in charge of its implementation as the main element for the success of this activity. Several reports on surgical campaigns have mentioned the low involvement of local authorities, including health authorities, in the planning of surgical campaigns, which has had a negative impact on the success of these campaigns, including: the interference of activities with its corollary on the increase in the workload of personnel of the local health system; the demobilization of routine health service providers; the late communication of the campaign timetable leading to low mobilization of communities; the shortcomings in the preparations (omissions of surgical materials, delay in the start of the campaigns in certain health centres due to the lack of prior arrangement of operating rooms, etc.); and the clashing of the campaigns with periods of agricultural and mining activities of the communities. However, this situation seems to have persisted, as shown in this study. Our data call on the personnel of the national program responsible for the fight against NTDs and their partners to work harder to adopt and promote micro-planning of trichiasis surgery activities, with the effective participation of personnel from endemic health districts. This would allow anticipating and possibly overcoming the specific challenges that arise in each district and community.

## 5. Conclusions

In conclusion, the main take away messages from this study are as follows. The most important obstacles to the effective implementation of TT surgery are essentially individual (the remoteness of surgery sites, medical costs associated with the services), community (fear of surgery, coincidence of campaigns with agricultural and mining activities), structural (inadequacy of health centres to provide surgical services, dilapidation and lack of hygiene in some places), and organizational (low involvement of local personnel in the planning of activities, limited timeframe of campaigns, insufficient logistical means for transporting patients and supervising activities). The adoption and promotion of micro-planning of activities and an integrated management of surgery including costs related to surgery (transport, food, support for the reintegration of operated patients) and to complications would be the first tasks of the national program personnel for the control of neglected tropical diseases to improve the coverage of the TT surgical management program.

## Figures and Tables

**Table 1:** Profile of respondents, N = 20.

Profile	Number (%)
Health system and services managers	8 (40)
Community Health Workers	4 (20)
Patients	7 * (35)
Community Leaders	1 (5)
**Gender**	
Male	16 (80)
Female	4 (20)

* 1 patient screened but not operated on for trachomatous trichiasis.

## Data Availability

The original contributions presented in this study are included in this article.
